# Informing phenomenological structural bone remodelling with a mechanistic poroelastic model

**DOI:** 10.1007/s10237-015-0735-4

**Published:** 2015-11-03

**Authors:** Claire C. Villette, Andrew T. M. Phillips

**Affiliations:** Structural Biomechanics, Department of Civil and Environment Engineering, Imperial College London, South Kensington Campus, London, SW7 2AZ UK; The Royal British Legion Centre for Blast Injury Studies at Imperial College London, South Kensington Campus, London, SW7 2AZ UK

**Keywords:** Mechanistic, Poroelastic, Remodelling, Bone, Phenomenological, Structural

## Abstract

Studies suggest that fluid motion in the extracellular space may be involved in the cellular mechanosensitivity at play in the bone tissue adaptation process. Previously, the authors developed a mesoscale predictive structural model of the femur using truss elements to represent trabecular bone, relying on a phenomenological strain-based bone adaptation algorithm. In order to introduce a response to bending and shear, the authors considered the use of beam elements, requiring a new formulation of the bone adaptation drivers. The primary goal of the study presented here was to isolate phenomenological drivers based on the results of a mechanistic approach to be used with a beam element representation of trabecular bone in mesoscale structural modelling. A single-beam model and a microscale poroelastic model of a single trabecula were developed. A mechanistic iterative adaptation algorithm was implemented based on fluid motion velocity through the bone matrix pores to predict the remodelled geometries of the poroelastic trabecula under 42 different loading scenarios. Regression analyses were used to correlate the changes in poroelastic trabecula thickness and orientation to the initial strain outputs of the beam model. Linear ($R^2>0.998$) and third-order polynomial ($R^2 >0.98$) relationships were found between change in cross section and axial strain at the central axis, and between beam reorientation and ratio of bending strain to axial strain, respectively. Implementing these relationships into the phenomenological predictive algorithm for the mesoscale structural femur has the potential to produce a model combining biofidelic structure and mechanical behaviour with computational efficiency.

## Introduction

It has long been observed that bone adapts its shape and structure to its mechanical environment (Wolff 1869; von Meyer 1867). The process involved in this functional adaptation is called bone remodelling and has been extensively studied (Frost 1987; Pioletti 2013). Bone tissue adaptation is a multi-aspect physiological process driven by interrelated mechanical and biological stimuli (Zadpoor 2013), which requires the combined activity of several populations of bone cells. Amongst them, the osteoclasts degrade bone material and the osteoblasts synthesise it (Rucci 2008). It is thought that osteoblast activity is triggered by signals sent by a third population of bone cells, the osteocytes (Burger and Klein-Nulend 1999; Temiyasathit and Jacobs 2010). The mechanism involved in the mechanosensitivity of osteocytes remains to be clarified (Rucci 2008).

Strains that are osteogenic at the tissue level (Rubin and Lanyon 1985) are below those which produce osteoblastic response to substrate deformation (You et al. 2000; Pereira and Shefelbine 2014), which tends to indicate that deformation of the solid matrix is not the sole trigger for mechanotransduction of the osteocytes attached to it. Studies suggest that fluid motion in the extracellular space of the lacunar-canalicular porosities where the osteocytes lay may be involved in cellular mechanosensitivity (Rubin et al. 2001; Qin et al. 2003; Cowin et al. 1995; Temiyasathit and Jacobs 2010), potentially via the resulting shear stress on the cell walls due to fluid motion (Adachi et al. 2010). A potential candidate as an extracellular sensor of mechanical loading is the primary cilium, a microtubule that protrudes from the cell membrane (Whitfield 2003; Temiyasathit and Jacobs 2010). In silico studies and simulations have implemented these theories in mechanistic models with probant results (Riddle and Donahue 2009; Adachi et al. 2010; Kameo and Adachi 2014; Pereira and Shefelbine 2014).

Extensive work has also been conducted using phenomenological approaches, based on the empirical relationships between mechanical stimulus and bone adaptation. Such approaches are limited in scale, due to the homogenisation of properties they assume, and in scope. Specifically, they do not allow direct investigation of the biological processes potentially involved in conditions affecting bone morphology such as osteoarthritis and osteoporosis. However, they present tremendous advantages in terms of model simplicity and computational efficiency. They have been used repeatedly in areas of biomechanics that focus on bone’s reaction to altered loading conditions such as fracture initiation (Hambli 2011), healing (Shefelbine et al. 2005) and the behaviour of bone-implant interfaces (Huiskes et al. 1987; Scannell and Prendergast 2009). Recent studies have focused on predicting bone structure entirely through bone adaptation considerations, based only on general information about the bone such as its surface geometry and its overall porosity value combined with loading data (Tsubota et al. 2009; Geraldes and Phillips 2010, 2014; Geraldes et al. 2015). While the results presented in these studies rely on continuum modelling, recent attempts have also combined structural modelling with bone adaptation predictions (Phillips et al. 2015; Phillips 2012; Marzban et al. 2013). Marzban et al. iteratively adapted the cellular structure of a 2D model of a proximal femur by adding or removing load-bearing elements in structural cells in order to reach a target stress. Results displayed some of the characteristic high-density trajectories observable in DEXA scans (Marzban et al. 2013).

In a previous study, the authors developed a mesoscale structural finite-element model of a femur relying on a phenomenological strain-based bone adaptation algorithm (Phillips et al. 2015). The study hypothesised that the structure of bones is optimised, in terms of amount and distribution of bone material, to withstand daily activities such as walking or going up and down the stairs. In the study, the surface geometry of a *Sawbones* surrogate was obtained from a CT scan, and the enclosed volume was meshed with tetrahedral elements of average edge length 4.5 mm. The surface faces of the elements were used to define homogeneously thin shell elements representing cortical bone. Each volume node was connected to its 16 closest neighbours to build a web of truss elements with homogeneous circular cross section to represent trabecular bone. This initial inner structure was considered randomised, as the truss distribution, orientation and cross sections were homogeneous. Loading conditions corresponding to joint contact forces, muscles forces, and inertia loading during daily activities were estimated using musculoskeletal simulations of gait cycles recorded on a volunteer (Modenese et al. 2011), and applied to the femur. The maximum absolute principal strain in the plane of the shells and the maximum absolute axial strain in the trusses over all the load cases were extracted and compared to a target range as proposed in Frost’s ‘Mechanostat’ (1987). The cross section of each element outside the target range was then linearly adapted with respect to the ratio of observed strain over target strain. This load application and subsequent adaptation were conducted iteratively until 99 % of the elements lay within the target range. The predicted structure showed a strong correlation with anatomical observations. Cortical thickness and trabecular density distribution were consistent with medical imaging, and the main proximal trabecular groups (primary compressive, primary tensile, secondary compressive, secondary tensile and greater trochanter tensile) described in the literature (Wolff 1869; von Meyer 1867) were reproduced. Computational efficiency was considered good, with a simple quasi-static loading scenario converging in under 3 min on a standard workstation. Such a predictive modelling approach does not require extensive clinical data, such as high-resolution CT scans which cannot be obtained in vivo. In addition, it has the potential to predict possible changes in bone morphology if a patient were to drastically change their daily loading activities or develop a condition which modifies their bone adaptation physiology. This modelling approach however presents limitations arising from the simplifications introduced in the truss formulation, where only axial strain is considered. In order to introduce a response to bending and shear, required for a complete description of the structural behaviour of bone, the authors considered the use of beam elements. This requires a new formulation of the bone adaptation drivers. In addition, it has been observed that bending-related loading scenarios lead to a reorientation of the structure, aligning to the trajectory of the load (Adachi et al. 2001, 2010; Kameo and Adachi 2014; Tsubota et al. 2009). The nodal repositioning involved is not supported in the authors’ current phenomelogical structural model.

The primary purpose of the study presented here was to isolate phenomenological drivers based on the results of a mechanistic approach, for future implementation in a strain-based bone adaptation algorithm to be used with a beam formulation of the trabecular elements in mesoscale structural modelling (Phillips et al. 2015). Following the concept developed by Adachi et al. (2010), the mechanistic approach should constitute a ‘framework of trabecular bone remodelling that interconnects the microscopic cellular activities to the macroscopic morphological changes through the mechanical hierarchy’. These microscale results would then be translated to the mesoscale model while maintaining its computational efficiency. With that aim, a continuum poroelastic model of a single trabecula was implemented utilising the fluid motion hypothesis (Adachi et al. 2010; Kameo and Adachi 2014; Pereira and Shefelbine 2014) for the bone remodelling mechanosensitivity pathway. This model was used to inform the phenomenological algorithm based on the assumption that the static structural model is able to capture the loading conditions corresponding to the initial state of the transient poroelastic simulation.

## Methods

### Overview

**Fig. 1:**
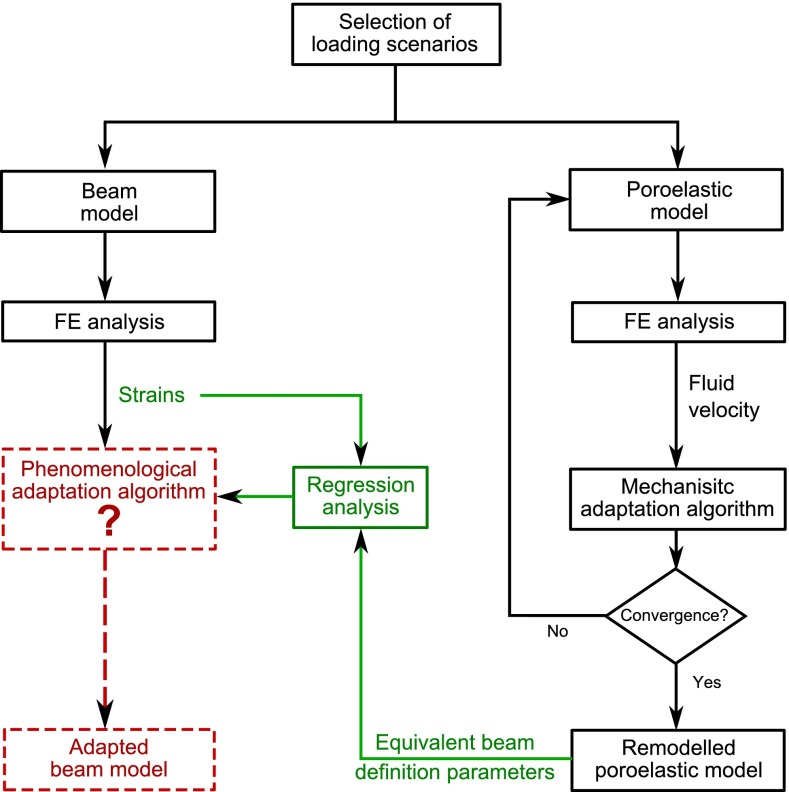
Flow chart of the study. In *black* are the simulation steps performed in this study. In *dashed red* are the simulation steps which will be made possible by the results of this study. In *green* are the analysis steps performed in this study, which will inform the red simulation steps, based on the results of the black simulations steps

The overall framework of the study is represented in the flowchart displayed in Fig. 1. The aim of this study was to derive the rules of a structural strain-based phenomenological adaptation algorithm. Hence, two distinct 2D finite-element models of a single trabecula were developed. In one model, the trabecula was represented by a single-beam element. In the other model, the trabecula was represented by a mesh of poroelastic elements. Both models were designed to represent equivalent set-ups and were subjected to the same selection of loading scenarios. An adaptation algorithm was implemented to iteratively remodel the poroelastic trabecula under loading, based on fluid motion velocity through the bone matrix pores. Even though it does not capture cellular mechanisms or biochemical responses, this algorithm will be referred to as ‘mechanistic’ to highlight the deeper level in the mechanotransduction hierarchy included in the modelling compared to the structural phenomenological adaptation algorithm. After convergence of this iterative process, the definition parameters of a reorientated beam equivalent to the continuum remodelled poroelastic trabecula were estimated for each load case. A regression analysis was conducted to derive relationships between the beam initial strains and the definition parameters of its adapted configuration.

### Initial models

#### Beam model

**Fig. 2:**
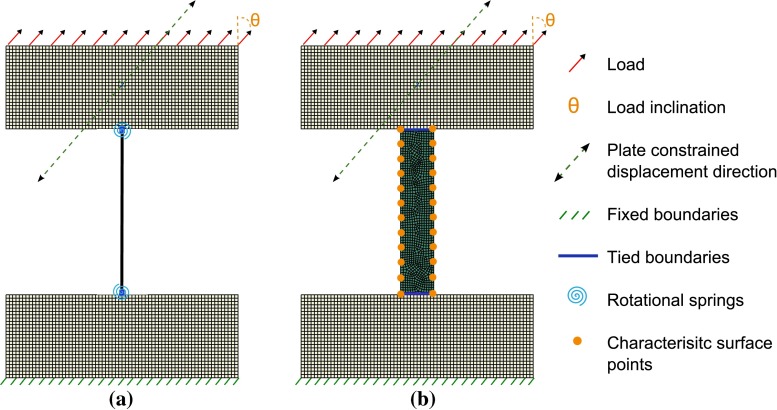
Simulation set-up. **a** Beam model. **b** Poroelastic model

A single three-nodded quadratic Timoshenko 2D beam element was used [*Abaqus 6.12* element B22 (Dassault Systemes 2013)]. It was assigned a circular cross section of radius 0.1 mm, a length of 1 mm and linear elastic isotropic properties consistent with the literature ($$E= 18 $$ kN / mm$$^2$$ and $$\nu = 0.3,$$ (Phillips 2012; Turner et al. 1999)). It was tied at both extremities to 0.5 mm thick, 1 mm deep and 1.4 mm wide plates with bone material properties, meshed with four-nodded linear plane stress continuum elements (CPS4R). Figure 2a shows the initial set-up with the boundary conditions and the points of load application. The intermediary node of the beam element was taken as the middle point between the top and the bottom nodes. The continuum elements constituting the plates do not have a rotational degree of freedom. Hence, the tied conditions applied did not prevent rotation of the beam with respect to the plate. This rotation was naturally constrained in the poroelastic model presented in Sect. 2.2.2 because the tied conditions were defined between edges, hence involving several pairs of tied nodes in parallel. To model this rotational constraint in the case of a beam, a rotational spring was added between the top node of the beam and the ground and its bottom node and the ground. The order of magnitude of the rotational spring stiffness was estimated to reproduce the stiffness provided by the bone plates in the poroelastic model. It was computed using the stiffness matrix coefficient for a beam element with the dimensions of the bottom plate.

$$\begin{aligned} k=\frac{4EI}{t} \end{aligned}$$

with *I* the second moment of area of the plate cross section, $t=0.5$ mm the plate thickness, $d=1$ mm the plate depth perpendicular to the model plane and $w=1.4$ mm the plate width

$$\begin{aligned} I=\frac{dw^3}{12} \end{aligned}$$

This yielded a rotational spring stiffness $$k=30 $$ kN mm.

#### Poroelastic model

Bone can be considered as a biphasic or poroelastic material with fluid present within the porous solid matrix (Cowin 1999). Poroelastic finite-element formulations are commonly used to predict pore pressure and fluid velocities in the lacunar-canalicular porosities (Pereira and Shefelbine 2014; Kameo and Adachi 2013, 2014; Adachi et al. 2010; Cowin et al. 1995). For a complete description of the poroelastic theory, the reader may refer to Biot (1941) and Detournay and Cheng (1993). Briefly, the poroelasticity theory describes the condition of the medium at a scale larger than the size of the pores so that it may be treated as homogeneous, and at the same time smaller than the region under consideration so that one element may be considered as infinitesimal in the numerical treatment. The average stress conditions in the medium must then satisfy the equilibrium equations of a stress field. These stresses can be interpreted as composed of two parts: one caused by the hydrostatic pressure of the fluid filling the pores, and the other caused by the average stress in the solid phase. An additional variable $\zeta$ accounting for the variation in fluid content must be considered in order to describe completely the macroscopic condition of the medium, with pore pressure *p* as its counterpart. If the changes in the medium are assumed to occur by reversible processes, the macroscopic condition of the medium described by the strain variables $\varepsilon _{ij}$ and the variation in fluid content must be a definite function of the stresses $\sigma _{ij}$ and the fluid pressure. For small quantities, these relationships can be considered as linear. If the medium is considered isotropic, the constitutive equations for the medium can then be expressed as follows:

$$\begin{aligned} \sigma _{ij}&=2G\varepsilon _{ij}+\left( \frac{2G\nu }{1}-2\nu \right) \varepsilon _{kk}\delta _{ij}-{\alpha }M{\zeta }\delta _{ij}\\ p&=M(\zeta -{\alpha }{\varepsilon _{kk}}) \end{aligned}$$

where i and j are tensor components, $\sigma _{ij}$ the stress tensor, $\varepsilon _{ij}$ the strain tensor, $\nu$ the drained Poisson’s ratio, *G* the drained shear modulus related to Young’s modulus E and $\nu$, $\alpha$ the Biot effective stress coefficient and *M* a coefficient such as:

$$\begin{aligned} \alpha&=1-\frac{K}{K_{s}}\\ M&=\frac{\phi }{K_f}+\frac{\alpha -\phi }{K_s} \end{aligned}$$

where $\phi$ is the porosity and *K*, $K_{s}$ and $K_f$ are, respectively, the drained bulk modulus, the solid matrix bulk modulus and the bulk modulus of the fluid phase. The field equations to be solved in the poroelastic problem can then be obtained from these constitutive equations using equilibrium considerations, compatibility equations for strain, and a continuity equation for the fluid phase which accounts for the mass conservation of a compressible fluid. This last equation relies on a transport law, such as Darcy’s law:

$$\begin{aligned} q=-\frac{\kappa }{\mu }\nabla _{p} \end{aligned}$$

where *q* is the fluid flux, $\kappa$ the intrinsic permeability, $\mu$ the interstitial fluid viscosity and $\nabla _{p}$ the gradient of pore pressure.

The single trabecula was modelled as a rectangle (cylinder projected on the 2D plane) with length 1 mm and width 0.2 mm. It was meshed using four-nodded linear plane strain poroelastic elements with characteristic element edge length of 0.015 mm (*Abaqus 6.12* element CPE4P (Dassault Systemes 2013)). This mesh refinement was validated based on a mesh convergence study. It was tied at both extremities to the same plates as described for the beam model. The elements were assigned fluid-saturated isotropic material properties. The solid bone matrix was assigned the same elastic properties as the beam model. The solid and fluid phases were assumed compressible with respective compressibilities of $K_s$=20 kN/mm$^2$ and $K_f$=2.3 kN/mm$^2$, and a pore volume fraction $\phi$ of 5 % (Pereira and Shefelbine 2014; Adachi et al. 2010; Kameo and Adachi 2014; Cowin et al. 1995). Viscosity $\mu$ and specific weight were adapted from Pereira and Shefelbine (2014) and Adachi et al. (2010) and set to 10$^{-9}$ Ns/mm$^{2}$ and 9.8$\times 10^{-6}$ N/mm$^3$ respectively, similar to the properties of salt water. Permeability was taken as 10$^{-20}$ m$^{2}$ (Pereira and Shefelbine 2014; Adachi et al. 2010; Beno et al. 2006).

Free-flow boundary conditions were applied to the two lateral edges of the trabecula by setting their pore pressure to zero, while fluid flow was prevented at the junction with the bone plates. Figure 2b shows this initial set-up.

### Parameters of interest

**Fig. 3:**
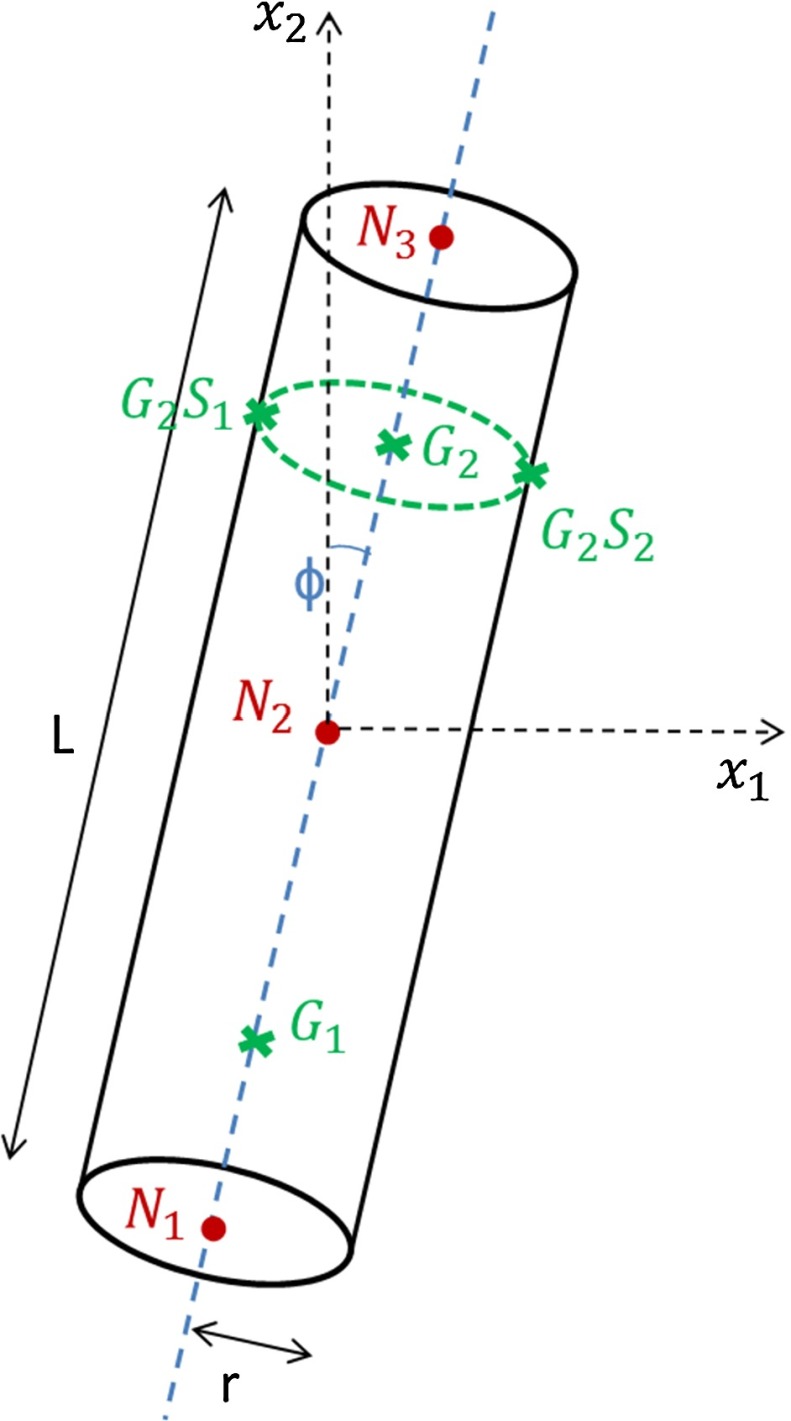
Schematic of the beam element parameters definition

For the beam element used, the normal strain in the direction of the beam is computed for a series of points. For each of the two integration points of the 2D beam element, strain values are computed at the central axis as well as a selection of section points through the cross section. In this study, the beam element is taken to have a constant circular cross section, which means that it can be completely defined by the spacial coordinates of three nodes and its cross-sectional area. As the intermediate node of the beam is taken as the middle point between the start and end nodes, the number of definition parameters is reduced to three: position of the middle node X$_{N2}$, inclination of the beam with respect to the vertical axis $x_{2}$, $\varphi$, and cross-sectional area $A=\pi r^2$. The parameters of interest are displayed on Fig. 3. Previous in silico work on trabecular remodelling has shown adaptation (thickening/thinning) in response to varying load amplitude and reorientation of the bone elements in the direction of the load (Adachi et al. 2001, 2010; Tsubota et al. 2009; Kameo and Adachi 2014). This effectively encourages longitudinal tension or compression deformation modes at the expense of bending. A good marker of bending in a beam element is the relative difference $\varepsilon _{b}$ in normal strain between opposite points on the outer surface of the cross section. Similarly, the normal strain at the central axis $\varepsilon _{a}$ provides information on the axial state (compression/tension) of the element. The ratio of the two $K_{\varepsilon }$ provides an indication of the preponderance of bending moment over axial force. The aim of this study was thus to isolate two relationships between beam strain outputs in the initial beam configuration (subscript ‘i’) and the parameters required to define the final adapted beam configuration ( subscript ‘f’) . More specifically, the two relationships sought would ideally predict the change in beam inclination $\Delta \varphi$ and the ratio of the beam initial and adapted cross-sectional areas $R_\mathrm{A}$ based on the bending strain and axial strain values obtained from the initial configuration. As the poroelastic trabecula model is two dimensional, with a constant depth, the ratio of the three-dimensional beam initial and adapted cross-sectional areas is equivalent to the ratio of the two-dimensional poroelastic trabecula initial and adapted widths $w_i$ and $w_f$. In a first version of the model presented here, the final position of node *N*2 X$_{N2f}$ is equal to the initial position of node *N*2, X$_{N2i}$ as the trabecular element would be expected to change in cross section and orientation but not in position. The strain outputs were taken at the second Gauss point. Based on the notation defined in Fig. 3, the strain and beam definition parameters are thus defined as follows, where all the strain outputs are extracted from the initial beam model:

$$\begin{aligned} \varepsilon _{b}&=\varepsilon _{G_2S_2}-\varepsilon _{G_2S_1}\\ \varepsilon _{a}&=\varepsilon _{G_2}\\ K_{\varepsilon }&=\dfrac{\varepsilon _{b}}{\varepsilon _{a}}\\ \Delta \varphi&=\varphi _f-\varphi _i \\ R_{A}&=\dfrac{w_f}{w_i} \end{aligned}$$

The primary aim of this study was thus to isolate two relationships *f* and *g*:

1 $$\begin{aligned}&\Delta \varphi =f(K_{\varepsilon }) \end{aligned}$$

2 $$\begin{aligned}&R_{\mathrm{A}}=g(\varepsilon _{a}) \end{aligned}$$

### Finite-element analyses

#### Simulation set-ups

The poroelastic simulations were conducted using the transient soil analysis option in *Abaqus (Standard)*, with a time step of one second. The loading was applied, in step increments of 0.1 s, linearly from zero to the amplitude prescribed in the plan of analysis over half of the time step and then decreased linearly over the other half. This load rate was consistent with the literature (Pereira and Shefelbine 2014; Adachi et al. 2010). The beam model simulations were run in a static analysis.

For both the beam model and the poroelastic model, the bottom edge of the bottom plate was fixed. The loading was evenly applied to the nodes at the top edge of the top plate. The top plate was constrained to displace only in the direction of the load, to maintain a constant load direction on the trabecula and avoid deformation of the plate in ways that could impact the load profile on the trabecula. A schematic of both set-ups is displayed in Fig. 2.

#### Plan of analyses

Five values of load amplitude *F* were selected in the range $[0.5F_{\mathrm{target}}, 2F_{\mathrm{target}}]$. Thirteen values of load inclination $\theta$ with respect to the vertical direction were selected between 0 and $\pi$. It was considered that results for the other half quadrant could be inferred by symmetry. The combinations of parameters tested are listed with a symbol ‘$\bullet$’ in Table 1.

**Table 1:** Loading scenarios tested

Angle	$F_{\mathrm{target}}/2$	$3F_{\mathrm{target}}/4$	$F_{\mathrm{target}}$	$3F_{\mathrm{target}}/2$	$2F_{\mathrm{target}}$
0	$\bullet$		$\bullet$		$\bullet$
$1\pi /16$		$\bullet$	$\bullet$	$\bullet$	
$2\pi /16$		$\bullet$	$\bullet$	$\bullet$	
$3\pi /16$		$\bullet$	$\bullet$	$\bullet$	
$\pi /4$	$\bullet$		$\bullet$		$\bullet$
$5\pi /16$		$\bullet$	$\bullet$	$\bullet$	
$6\pi /16$		$\bullet$	$\bullet$	$\bullet$	
$7\pi /16$		$\bullet$	$\bullet$	$\bullet$	
$\pi /2$	$\bullet$		$\bullet$		$\bullet$
$9\pi /16$		$\bullet$	$\bullet$	$\bullet$	
$10\pi /16$		$\bullet$	$\bullet$	$\bullet$	
$3\pi /4$	$\bullet$		$\bullet$		$\bullet$
$14\pi /16$		$\bullet$	$\bullet$	$\bullet$	
$\pi$	$\bullet$		$\bullet$		$\bullet$

### Poroelastic pipeline

#### Mechanistic remodelling algorithm

In this study, the mechanistic remodelling algorithm was based on the fluid motion theory. Hence, pore fluid velocity, directly linked to cell membrane shear stress and strain, was chosen as the driver for bone apposition or resorption. This driver is consistent with previous in silico work. For instance, Adachi et al. (2010) used membrane shear stress and Pereira and Shefelbine (2014) used the integration of absolute fluid velocity over the time step.

Just as in the previous studies, a target fluid velocity range was chosen. Its limits can be interpreted as thresholds for cell mechanosensitivity. They were obtained through a trial-and-error calibration: A purely compressive force $F_{\mathrm{target}}=A\times \varepsilon _{\mathrm{target}}$ was applied to the model, with $\varepsilon _{\mathrm{target}}=1250\mu \varepsilon$ the target strain proposed by Frost (1987) and *A* the cross-sectional area of a cylindrical trabecula with radius equal to half the width of the 2D trabecular model in this study. According to the ‘Mechanostat’ principle, such a load case should elicit minimal adaptation. The remodelling algorithm was run with varying fluid velocity targets and target range limits until ‘qualitatively minimal’ remodelling was obtained. The target values were thus set to $V_{\mathrm{target}}=5\times 10^{-8}$ mm / s, $V_{\mathrm{apposition}}=5.2\times 10^{-8}$ mm/s and $V_{\mathrm{resorption}}=4.8\times 10^{-8}$ mm/s, with $V_{\mathrm{apposition}}$ and $V_{\mathrm{resorption}}$ the upper and lower limits of the target range, respectively, outside which bone remodelling will occur.

Remodelling happens on the surface of the bone elements (Rucci 2008). In this study, twelve points were chosen uniformly distributed on each of the two lateral sides of the initial trabecula model to assess the distribution of fluid velocity (cellular activity), as displayed in Fig. 2b. The median fluid velocities of all poroelastic elements located in a sphere of influence around each point were averaged to determine a characteristic fluid velocity *V* at that point. The median was chosen instead of the mean to minimise potential errors arising from outliers and finite-element edge effects. The radius of the sphere of influence was taken as a third of the distance between two characteristic points. An updated horizontal component of the position $Xp_{n+1}$ was computed for each of these points *p*:

3 $$\begin{aligned} Xp_{n+1}=Xp_n \pm \mathrm {min}\left( 0.005\times \dfrac{V}{V_{\mathrm{target}}},0.05\right) \end{aligned}$$

Where 0.05 corresponds to the maximum allowable displacement in an iteration, in mm, and 0.005 to an arbitrary displacement increment in mm. The value of $\pm$ depended on which side was considered and whether bone material was deposited ($V\ge {V_{\mathrm{apposition}}}$) or resorbed ($V\le {V_{\mathrm{resorption}}}$). The adapted trabecular geometry was then generated between two splines passing through the updated position of these 24 points and two straight lines joining the top points of both splines and the bottom points of both splines. This planar model was then automatically remeshed to produce the updated model. This topology adaptation was conducted iteratively for 100 iterations. At that point, no characteristic point showed a displacement greater than 5 % of the trabecula central width and the adaptation was considered complete. The central width was approximated as the difference in horizontal position at the middle characteristic points. The pipeline was entirely automated, using *Python* scripts to extract information from an *Abaqus* output database and generate new *Abaqus* models, and *MATLAB* to compute the updated characteristic point positions.

#### Poroelastic pipeline validation test

The poroelastic remodelling pipeline was tested on a scenario adapted from Adachi et al. (2010) for comparison of results: A 0.2 mm wide and 0.92 mm long trabecula with the material properties described above was modelled with an incline of 30$$^{\circ }$$ with respect to the vertical. Its bottom edge was constrained in the vertical direction. For consistency with the simulation described in Adachi et al. (2010), pore fluid flow was set free on all edges, including the junctions with the bone plates. Its upper edge was in hard contact with a stiff isotropic elastic plate ($$E=200 $$ kN/mm$$^2$$, $$\nu =$$0.3) constrained in all degrees of freedom but the vertical displacement. A pressure of 0.4 N/mm$$^2$$ was linearly applied to the top edge of the plate over the 1 s long transient soil analysis step.

### Model correlation

The definition parameters of beams equivalent to the remodelled poroelastic trabeculae were estimated for all the loading scenarios tested. The position of the start and end nodes were taken as the middle points between the left and right extremities of the corresponding edge of the trabecula models. The inclination $\varphi _{f}$ was taken as the scalar product of the vector defined by these two nodes and the unit vertical vector. The initial inclination being zero, the change in beam inclination $\Delta \varphi$ was taken as the inclination of the adapted beam model $\varphi _{f}$.

An apparent trabecular radius was estimated as half of the mean between the differences in horizontal position of the characteristic points of both splines. The remodelled radius was computed as the projection of the apparent radius over the direction perpendicular to the beam axis defined by the start and end nodes. The changes in the beam definition parameters between initial and equivalent adapted configurations ($\Delta \varphi$ and $R_{\mathrm{A}}$) were computed and plotted against the strain outputs from the structural model ($K_{\varepsilon }$ and $\varepsilon _{a}$) for all corresponding loading scenarios. Relationships between both sets of parameters were then found by regression and assessed for goodness of fit.

## Results

### Validation of the poroelastic model

**Fig. 4:**
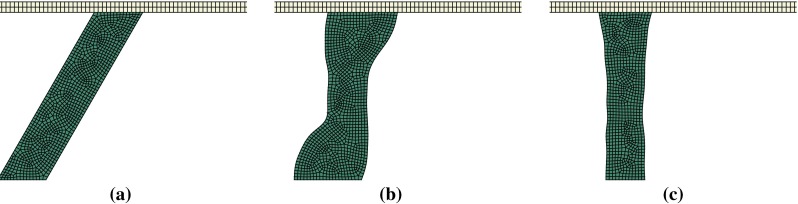
Evolution of the trabecula geometry during the validation test. **a** Initial iteration. **b** Iteration 40. **c** Iteration 100 (converged)

Figure 4 shows the evolution of the trabecular morphology during the validation test over 100 iterations of the remodelling algorithm. It results in a complete reorientation in the vertical direction (the direction of the load) as well as a thinning of about 20 % of the apparent cross section. This is consistent with the results presented by Adachi et al. (2010).

### Remodelling of the poroelastic trabeculae

**Fig. 5:**
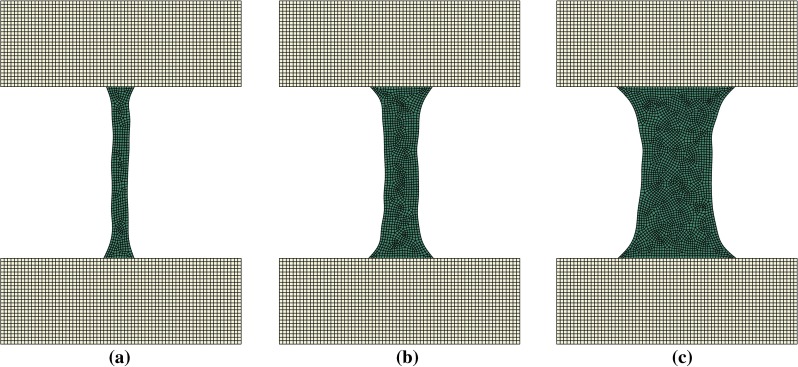
Adapted shape of trabeculae remodelled under a load application angle of $\pi$ rad at three different load amplitudes **a** $0.5F_{\mathrm{target}}$. **b** $F_{\mathrm{target}}$. **c** $2F_{\mathrm{target}}$

**Fig. 6:**
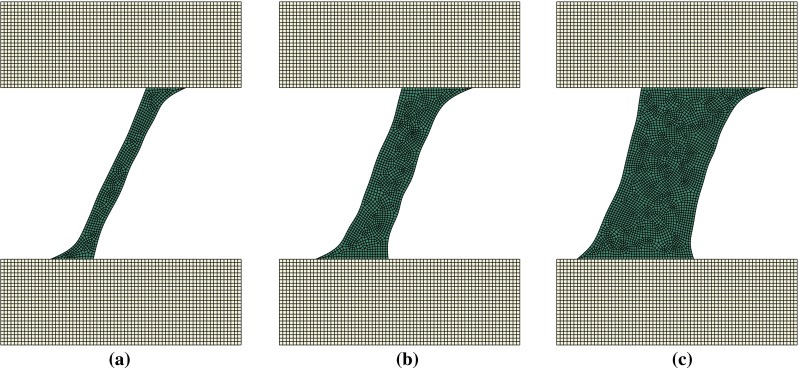
Adapted shape of trabeculae remodelled under a load application angle of $\pi /4$ rad at three different load amplitudes **a** $0.5F_{\mathrm{target}}$. **b** $F_{\mathrm{target}}$. **c** $2F_{\mathrm{target}}$

**Fig. 7:**
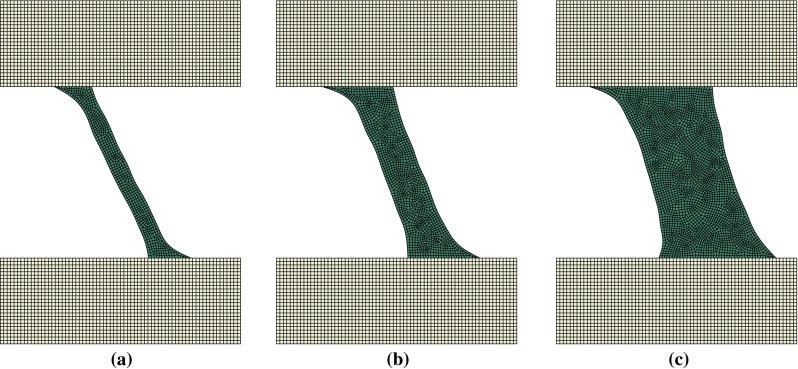
Adapted shape of trabeculae remodelled under a load application angle of $3\pi /4$ rad at three different load amplitudes **a** $0.5F_{\mathrm{target}}$. **b** $F_{\mathrm{target}}$. **c** $2F_{\mathrm{target}}$

Figures 5, 6 and 7 display the remodelled trabecula geometries for the loading scenarios obtained by combinations of $0.5{F_{\mathrm{target}}}$, $F_{\mathrm{target}}$ and $2 F_{\mathrm{target}}$ with inclination angles of $\pi$, $\pi /4$ and $3\pi /4$, respectively.

**Fig. 8:**
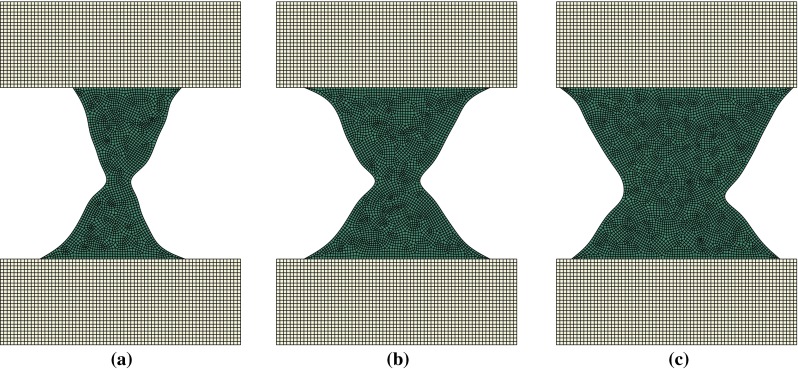
Adapted shape of trabeculae remodelled under a load application angle of $\pi /2$ at three different amplitudes **a** $0.5F_{\mathrm{target}}$. **b** $F_{\mathrm{target}}$. **c** $2F_{\mathrm{target}}$

Figure 8 displays the remodelled trabecula geometries for the purely lateral (inclination of $\pi /2$) loading scenarios with amplitudes $0.5{F_{\mathrm{target}}}$, $F_{\mathrm{target}}$ and $2 F_{\mathrm{target}}$. Trabecular adaptations for all other angles of load application result in configurations similar to those displayed in Figs. 5,  6 and 7. Mean width increases with load amplitude, and the orientation of the trabecula axis varies consistently with the direction of the load.

### Model correlation

**Fig. 9:**
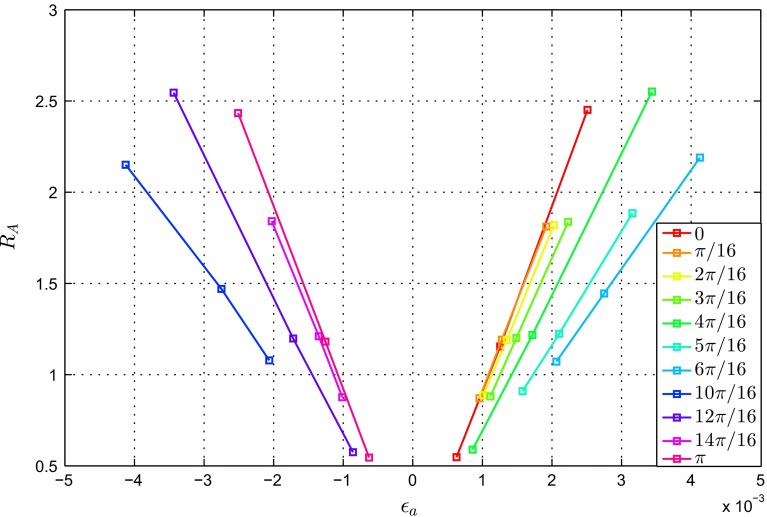
Ratio of the beam initial and adapted cross-sectional areas $R_{\mathrm{A}}$ against the normal strain at the central axis $\varepsilon _{a}$

**Fig. 10:**
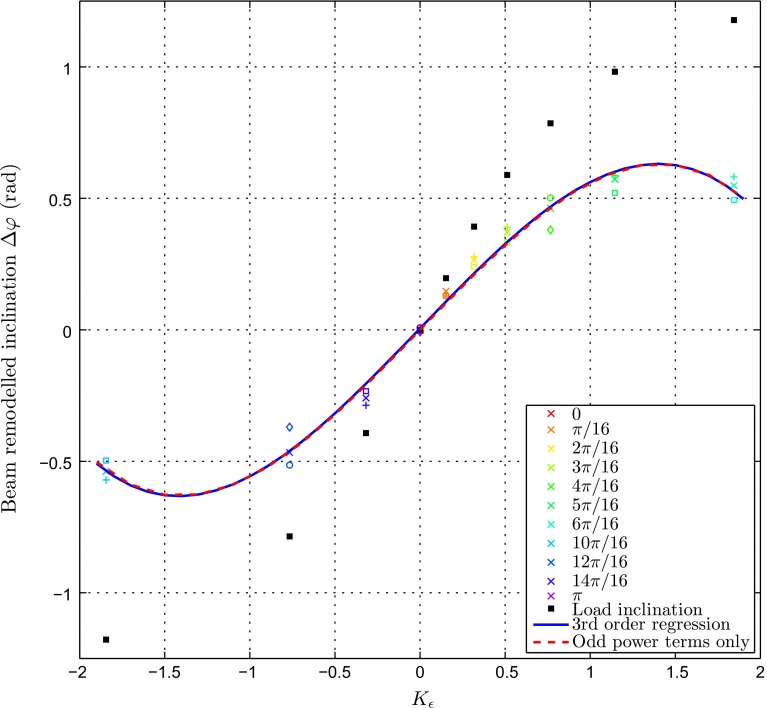
Change in beam inclination $\Delta \varphi$ against the ratio of bending strain over normal strain at the central axis $K_{\varepsilon }$. 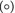 $$F_{\mathrm{target}}/2$$, 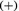 $$3F_{\mathrm{target}}/4$$, 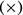 $$F_{\mathrm{target}}$$, 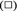 $$3F_{\mathrm{target}}/2$$, 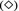 $$2F_{target}$$

Figures 9 and 10 show the changes in the beam definition parameters ($\Delta \varphi$, and $R_{\mathrm{A}}$) plotted against the strain outputs $K_{\varepsilon }$ and $\varepsilon _{a}$ for the loading scenarios with $\theta$ in the range $[0, 6\pi /16] \cup [10\pi /16, \pi ]$.

**Fig. 11:**
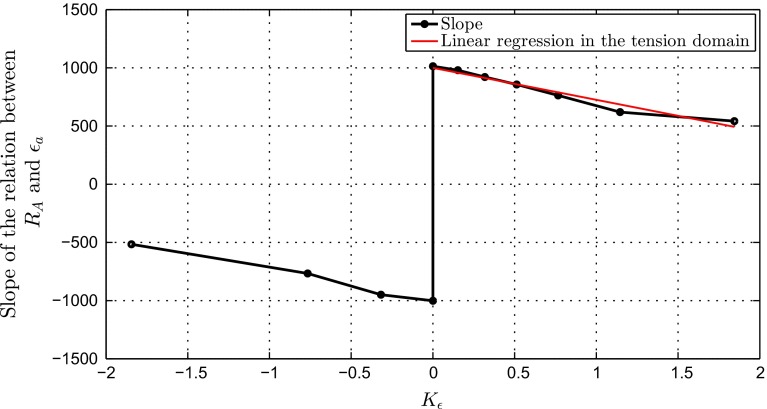
Slopes of the linear relationships between the ratio of the beam initial and adapted cross-sectional areas $R_{\mathrm{A}}$ and the normal strain at the central axis $\varepsilon _{a}$ as a function of the ratio of bending strain over strain at the central axis $K_{\varepsilon }$

The results shown in Fig. 9 present a family of curves relating $R_{\mathrm{A}}$ to $\varepsilon _{a}$. For all angles of application of the load $\theta$, $R_{\mathrm{A}}$ increases linearly with $\varepsilon _{a}$ ($R^{2}>0.998$). The slope of the linear fit increases with $\theta$. Further description of this trend is provided in the Discussion and in Fig. 11.

$\Delta \varphi$ increases with $K_{\varepsilon }$. For $\theta \in [0, 5\pi /16] \cup [11\pi /16, \pi ]$, the relationship is quasi-linear ($R^{2}> 0.96$). However, the rate of change in $\Delta \varphi$ decreases significantly with $\theta$ close to the horizontal. A third-order polynomial provided a good fit for the relationship over the entire design space ($R^{2}> 0.98$) and is displayed in black in Fig. 10. Its equation is given below:

$$\begin{aligned} P(x)=-0.1129x^3-0.003x^2+0.6725x+0.0054 \end{aligned}$$

The coefficients of even powers are two orders of magnitude lower than the coefficients of odd powers. It would thus be sensible to ignore the even power terms. This choice is further justified as a symmetrical relationship between $\Delta \varphi$ and $K_{\varepsilon }$ would be expected, as two loading scenarios equivalent but for the direction of bending would yield $K_{\varepsilon }$ of the same magnitude, while likely reorienting by the same angle in opposite directions. The plot displayed in Fig. 10 supports this remark. The polynomial ignoring even power terms is plotted in red in Fig. 10. As a result, it is possible to model the relationship between $\Delta \varphi$ and $K_{\varepsilon }$ as described in Eq. 4.

4 $$\begin{aligned} \Delta \varphi =aK_{\varepsilon }^{3}+bK_{\varepsilon } \end{aligned}$$

with $$a=-0.1129, b=0.6725$$

## Discussion

For all the loading scenarios tested, including pure tension and pure compression at force amplitude $F_{target}$, the remodelled trabecula geometry changed from the initial perfect rectangle to a rod-like shape with wider extremities and a thinner central cross section. This is consistent with $\mu$CT observation of individual trabeculae (Van Lenthe et al. 2006).

The calibration of the target fluid velocities was arbitrarily assigned, based on achieving a match with the well-established strain targets proposed by Frost (1987), and further work would benefit from investigating an optimisation process more directly related to the mechanotransductive process. The peak fluid velocities observed in this study were of the order of magnitude of 10$^{-5}$mm/s. This is consistent with previous computational work (Pereira and Shefelbine 2014; Fornells et al. 2007). Based on experimental measurements for similar loading rates and amplitudes, Price et al. (2011) estimated peak fluid flow velocities of 60$\mu$ m/s. The values predicted in this study are well below this estimation. Poroelasticity theory computes averaged quantities, such as fluid velocities, over the element volume; hence, an underestimation of peak quantities is expected. It is, however, unclear why such a large difference is observed, although tortuosity and directionality of the fluid phase network might influence this phenomenon.

Adaptation in tension ($\theta < \pi /2$) and compression ($\theta > \pi /2$) scenarios yielded near-symmetrical results. This was to be expected considering that the driver considered for the poroelastic remodelling algorithm was the absolute value of the fluid velocity, which did not distinguish between inward fluid motion generated in tension and outward fluid motion generated in compression. Potential physiological justifications for a different cellular reaction according to the direction of the fluid motion are of future interest but were considered outside the scope of this study.

The trabecula orientations adapted consistently with the load inclination; however, they did not match them exactly. The offset can be visualised on Fig. 10, where the direction of load application was plotted against $K_{\varepsilon }$. It is interesting to note that this offset increases with load amplitude. As further refinement, a correcting coefficient could be estimated and included into the relationship *f* between $\Delta \varphi$ and $K_{\varepsilon }$ to account for this influence of load magnitude on the adapted trabecula orientation.

The offset, and its increase with load amplitude are very likely due to the non-zero width of the target range, as a less precise alignment of $\Delta \varphi$ with $\theta$ would be required for a low load amplitude to stay within the target range. The boundary conditions of the top plate whose displacement is constrained generate reaction forces which are then transmitted to the trabecula, and might influence the direction of the locally applied load (overall equilibrium maintained and the sum of all reactions compensate the load applied).

The interpretation of the results of bone remodelling to a purely lateral load is unclear. It seems like a combination of the adaptation to a load with a small positive $\theta$ and a small negative $\theta$. One could imagine that in a 3D scenario, the central part of this geometry would resorb, leaving two rod-like elements inclined by a small angle with respect to horizontal. For the angles closest to the horizontal $\theta =7\pi /16$ and $\theta =9\pi /16$, a similar behaviour, although to a much lower extent, was observed.

For all other $\theta$, the adapted cross section can be approximated using a linear relationship with $\varepsilon _{a}$. The constant term *k* of these linear relationships varies between $-0.1$ and $-0.02$. It is not exactly zero due to the non-zero width of the target range. In the design space chosen, fixing the constant to the average $k_{mean}=-0.065$ will not generate errors higher than 10 %. Setting it to zero might be a way of representing a scenario of near-zero width target range. For these relationships to be used within a phenomenological algorithm, where only the beam strains would be outputted, one needs to be able to predict the slope of the linear relationship. Figure 11 shows the evolution of the slope of the linear relationship between $R_{\mathrm{A}}$ and $\varepsilon _{a}$ as a function of $K_{\varepsilon }$. This relationship is continuous except at the point $K_{\varepsilon }=0$. It is symmetrical between tension and compression scenarios: the slope amplitude is given by the preponderance of bending, while its sign is given by the sign of $\varepsilon _{a}$. In the subdomain corresponding to tension, the relationship between the slope and $K_{\varepsilon }$ can be approximated by a linear function with $R^{2}>0.95$. The corresponding linear fit is displayed in Fig. 11. Due to the symmetry mentioned above, a similar linear relationship can be derived between the slope and $K_{\varepsilon }$ in the compression subdomain, with the negative of the constant. From these concepts, it is possible to build a complete definition of the relationship between $R_{\mathrm{A}}$, $\varepsilon _{a}$ and $K_{\varepsilon }$, detailed in Eq. 5.

5a $$\begin{aligned}&R_{\mathrm{A}}=g(\varepsilon _{a},K_{\varepsilon }) \end{aligned}$$

5b $$\begin{aligned}&g(\varepsilon _{a},K_{\varepsilon })= h(\varepsilon _{a},K_{\varepsilon }) \varepsilon _{a}+k_{\mathrm{mean}} \end{aligned}$$

5c $$\begin{aligned}&h(\varepsilon _{a},K_{\varepsilon })=iK_{\varepsilon }+\text {sign}(\varepsilon _{a})j \end{aligned}$$

which can be expressed in a compressed form

6 $$\begin{aligned} R_{\mathrm{A}}=(iK_{\varepsilon }+\text {sign}(\varepsilon _{a})j)\varepsilon _{a}+k_{\mathrm{mean}} \end{aligned}$$

with $$k_{\mathrm{mean}}=-0.065, i={-274.654}, j=999.7622$$

It should be noted that the adapted position of the centre of the trabecula was not computed. In a perfectly symmetrical set-up, it could be assumed that this point remains fixed, the trabecula ‘rotating’ around it. However, the set-up in this study only allows loading from above, and in reality, trabecular elements are likely not to be loaded symmetrically from both sides.

**Fig. 12:**
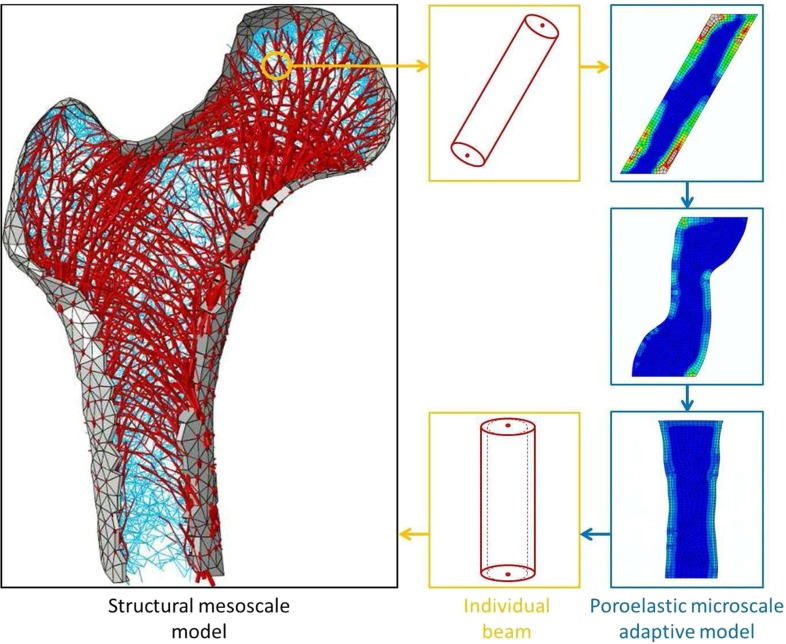
Schematic of the use of the relationships derived in this study within the phenomenological structural model developed previously (Phillips et al. 2015)

At this stage of development, the relationships derived can be implemented in the authors’ phenomenological structural model (Phillips et al. 2015; Phillips 2012). The concept of this implementation is depicted in Fig. 12. The proposed method would involve extracting the strain outputs for each individual beam element and computing its adapted cross section and its adapted inclination using equations (4) to (6). The adapted position of the top and bottom nodes defining each beam element would then be computed based on the element length and the inclination. Each node being potentially part of several elements, an average updated position weighted by the element cross sections should be considered. A significant limitation to this study is that it has been conducted in 2D and involved assumptions regarding the translation of the observations into a 3D problem. The authors’ structural model being in 3D, averaging of computations in three perpendicular planes should also be considered. A later stage of development would be to generalise the relationships derived to a 3D space. Similar relationships could also be derived for shell elements, using for instance the principal in-plane strains from the top and bottom surfaces of the elements. This 2D definition also impacted the cross-sectional properties (area and second moment of area, for example) of the elements: while the beam formulation was used with a circular cross section, a constant ‘through-plane’ depth was assigned to the poroelastic trabecula, effectively generating a rectangular cross section. This third-dimensional geometrical difference influenced the structural stiffness of the models. However, this structural stiffness difference was homogeneous, meaning that the orders of magnitude of the field variables were scaled consistently with the cross-sectional properties, but the profile of their spatial variation remained the same in both configurations. As the calibration process is based on detecting spatial relative differences, it typically cancels the influence of parameters with homogeneous effect on the model. Hence, despite yielding different field variables, both the beam model and its poroelastic counterpart retain their structural equivalence for the purpose of this 2D study. It is important to note that the authors’ structural mesoscale models are not intended to capture the transient phenomena related to the fluid phase. The capability to account for such phenomena is not retained in the beam models presented in this study, although the developed relationships will allow mesoscale beam models to be informed by the results of the microscale poroelastic models, based on the behaviour of the transient fluid phase.

Another limitation is that, to this point, no branching phenomenon has been implemented in the poroelastic model. It is, however, a potential mechanism of adaptation, already reported in in silico work (Kameo and Adachi 2013). This will also constitute a point of further work, in two and three dimensions.

To summarise, a single-beam model and a microscale poroelastic model of a single trabecula were developed. A mechanistic iterative adaptation algorithm was implemented based on fluid motion velocity through the bone matrix pores to predict the remodelled geometries of a single trabecula under 42 different axial loading scenarios. The remodelled geometries were used to derive two phenomenological relationships ($R^2>0.95$) to predict the beam changes in cross section and orientation from its initial strain outputs. It is expected that the implementation of these relationships into the phenomenological predictive algorithm for a full mesoscale femur modelled with beams and shells will produce a model whose structure and mechanical behaviour are biofidelic while maintaining the high computational efficiency of a purely structural model.

## List of symbols

$\varphi$: Inclination of the beam with respect to the vertical axis
$\Delta \varphi$: Change in beam inclination
*A*: Beam cross-sectional area
$R_\mathrm{A}$: Ratio of the beam initial and adapted cross-sectional areas
$\varepsilon _{\mathrm{b}}$: Relative difference in normal strain between diametrically opposite points on the outer surface of the beam cross section. Also referred to as ‘bending strain’ in this study.
$\varepsilon _{\mathrm{a}}$: Normal strain at the beam central axis
$K_{\varepsilon }$: Ratio of $\varepsilon _{b}$ over $\varepsilon _{a}$
*f*: Function defining the relationship between $\Delta \varphi$ and $K_{\varepsilon }$
*g*: Function defining the relationship between $R_A$ and $\varepsilon _{a}$
